# Interleukin-33 in Systemic Sclerosis: Expression and Pathogenesis

**DOI:** 10.3389/fimmu.2018.02663

**Published:** 2018-11-15

**Authors:** Liya Li, Honglin Zhu, Xiaoxia Zuo

**Affiliations:** ^1^Department of Rheumatology and immunology, Xiangya Hospital, Central South University, Changsha, China; ^2^The Institute of Rheumatology and Immunology, Central South University, Changsha, China

**Keywords:** interleukin-33, ST2, systemic sclerosis, pathogenesis, fibrosis

## Abstract

Interleukin-33 (IL-33), a member of the IL-1 superfamily, functions as a traditional cytokine and nuclear factor. It is proposed to have an “alarmin” role. IL-33 mediates biological effects by interacting with the ST2 receptor and IL-1 receptor accessory protein, particularly in innate immune cells and T helper 2 cells. Recent articles have described IL-33 as an emerging pro-fibrotic cytokine in the immune system as well as a novel potential target for systemic sclerosis. Here, we review the available information and focus on the pleiotropic expression and pathogenesis of IL-33 in systemic sclerosis, as well as the feasibility of using IL-33 in clinical applications.

## Introduction

Systemic sclerosis (scleroderma, SSc) is a heterogeneous autoimmune disease of unknown etiology, clinically characterized with obliterative microvasculopathy, inflammation, and extensive fibrosis of the skin and multiple organ systems and serologically characterized by the presence of circulating specific autoantibodies. SSc has the highest cause-specific mortality among connective tissue diseases (1, 2), and pulmonary artery hypertension and interstitial lung disease (ILD) are the leading causes of death (3, 4). Therapeutic interventions for SSc mainly involve the comprehensive administration of glucocorticoids and immunosuppressants and targeted treatment. To date, no effective medical intervention has been developed to control and reverse the progression of this fibrotic disease (5). Thus, effective and safe targeted therapies for SSc-related fibrosis are urgently needed. In the pathogenesis of SSc, endothelial damage may be a primary event. SSc also exhibits complex interactions during the transition from fibroblasts to myofibroblasts and non-infective inflammation or autoimmunity.

Interleukin (IL)-33 belongs to the IL-1 superfamily and is widely expressed throughout the human body. During cell damage or tissue injury, IL-33 is released into the extracellular space, wherein it produces endogenous danger signals to alert adjacent cells. This function deems IL-33 as an alarmin. IL-33 also functions as a nuclear factor regulating gene transcription in cytokine-expressing or cytokine-responsive cells (6). IL-33 is known to play crucial roles in inflammation. However, recent studies indicated that IL-33 participates in the development and progression of fibrotic diseases and SSc. Here, we review the profibrotic roles of IL-33 and its related mechanisms and discuss its potential application in the treatment of SSc.

## Biological characteristics of IL-33

IL-33, also known as IL-1F11, is a member of the IL-1 superfamily (7) and exhibits dual functionality (8). This cytokine was first identified as a nuclear factor in high endothelial venules in 2003 (9) but was renamed as IL-33 when a study in 2005 demonstrated its role as a specific extracellular ligand for the orphan IL-1 receptor family member ST2 (also known as IL-1RL1, DER4, T1, and FIT-1). ST2 is a member of the Toll-like receptor (TLR)/IL-1 receptor superfamily (10), which has two main isoforms, namely, a short soluble form (sST2) and longer transmembrane form (ST2L), with four isoforms in total, including ST2V and ST2LV (11). The mRNA encoding sST2 is a secretory sequence that is generated by alternative splicing and lacks the sequence encoding the transmembrane domain of ST2L (12).

**Graphical Abstract:**
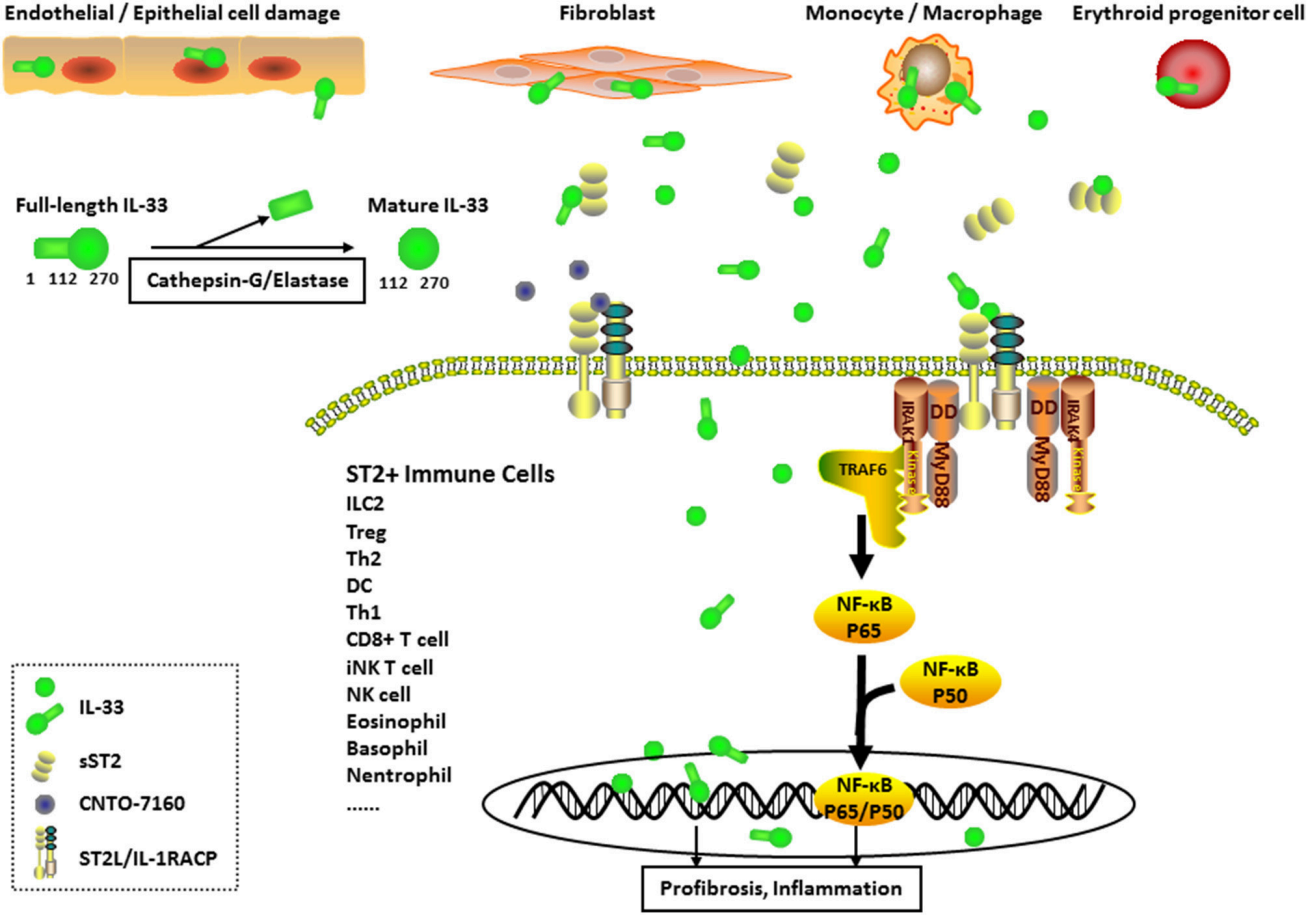
Expression and Pathogenesis of interleukin (IL)-33 in Systemic sclerosis (SSc). IL-33-producing cells (mainly endothelial cells, epithelial cells, fibroblasts, monocytes, macrophages, and erythroid progenitor cells) secrete IL-33 into the extracellular environment. Full-length IL-33 is transformed into mature IL-33 through cleavage by inflammatory proteases (such as cathepsin-G and elastase). The formation of the IL-33/ST2 longer transmembrane form (ST2L)/IL-1 receptor accessory protein complex in ST2+ immune cell membranes results in the activation of nuclear factor-κB transcription factors through the MyD88, IL-1R-associated kinase (IRAK), and tumor necrosis factor receptor associated factor 6 (TRAF6) signaling pathways, leading to the induction of inflammation and profibrosis in pathological cells. IL-33 also functions as a nuclear factor to regulate gene transcription in cytokine-expressing or cytokine-responsive cells. Moreover, sST2 acts as a decoy receptor for IL-33 (full-length IL-33 or mature IL-33), and CNTO-7160 (the first monoclonal anti-ST2 antibody) was designed as a new IL-33 inhibitor. Both of these molecules block the downstream signaling of IL-33.

The IL-33 gene is located on human chromosome 9 (or chromosome 19 in mice) and is transcribed from seven coding exons. The protein is synthesized as a 31-kDa pro-IL-33_1−−270_ (full-length IL-33). Following synthesis, IL-33 is transported into the nucleus as a nuclear factor. Similar to the IL-1 family members IL-1β and IL-18, IL-33 lacks the classic signal sequence necessary for the transport by the endoplasmic reticulum/Golgi secretion pathway (13). Upon natural secretion from pathological cells undergoing necrosis or necroptosis, the full-length IL-33 is cleaved by caspase-3 and caspase-7 to activate apoptotic pathways in the cytoplasm, followed by its release into the extracellular environment (14). Once released into the extracellular matrix, full-length IL-33 is further processed by serine proteases (such as cathepsin-G and elastase) into the 18-kDa IL-33_112−−270_ (mature IL-33) with increased activity (15, 16), forming a soluble recombinant cytokine in circulation. However, both full-length and mature IL-33 bind to ST2L in ST2^+^ immune cell membranes and interact with IL-1 receptor accessory protein (IL-1RAcP), eventually leading to the formation of an IL-33/ST2L/IL-1RAcP complex. This complex induces signaling pathways through MyD88, IL-1R-associated kinase (IRAK), and tumor necrosis factor receptor associated factor 6 (TRAF6) and activates the canonical nuclear factor-κB (NF-κB) and mitogen-activated protein kinase (MAPK) pathways (17).

IL-33 is principally produced by stromal cells, including epithelial cells, endothelial cells, fibroblast-like cells, and myofibroblasts of lymphoid as well as non-lymphoid organs, under both steady state and inflammation conditions (18–20). In erythroid progenitor cells, IL-33 is produced during the maturation of red blood cells and released upon haemolysis (21). Innate immune cells expressing ST2 mainly include dendritic cells (DCs), natural killer (NK) cells, eosinophils, basophils, macrophages, and neutrophils (12). Full-length IL-33 predominantly remains inside the cell and regulates the expression of genes, which induce pulmonary inflammation and fibrosis. In contrast, mature IL-33 promotes asthma as well as allergic and anti-parasitic responses through the ST2 receptor and Th2 mechanisms (22).

Mechanisms such as inactivation by oxidation of cysteine residues, nuclear localization or sequestration, and proteolytic processing, and receptor antagonists as well as sST2 have evolved to regulate the expression and activities of IL-33 (12, 14, 15, 23, 24). sST2 is constitutively expressed in the human serum, wherein it acts as a decoy receptor for IL-33 and is not involved in signaling (25, 26). sST2, induced during tissue damage, may restrict the deleterious effects of increased IL-33 level. A novel mechanism for the rapid inactivation of IL-33 protein released from the cell *in vivo* was reported, wherein an oxidation-driven conformational change involving the formation of two disulphide bonds was observed, resulting in the elimination of ST2-dependent activity and reduction of inflammation, consistent with the mechanism of many other IL-1 family members (23).

## Expression of IL-33 and ST2 in SSC

According to recent studies, increased IL-33 and sST2 levels have been observed in patients with infections, cardiovascular disorders, allergic diseases, and rheumatic diseases such as systemic lupus erythematosus, rheumatoid arthritis (RA), Wegener's granulomatosis, and Behcet's disease (27–31). The serum levels of sST2 and synovial fluid of IL-33 were higher in patients with RA than in healthy controls and patients with osteoarthritis (27). Furthermore, serum sST2 levels were higher in patients with active, newly diagnosed, anti-neutrophil cytoplasmic antibody-associated vasculitis than in patients in remission, indicative of the marker role for sST2 (32). Furthermore, ST2 and IL-33 were highly expressed around ectopic germinal centers in salivary glands from patients with IgG4-related disease, whereas IL-33 was expressed only in epithelial cells in patients with Sjögren's syndrome and controls (33). Interestingly, the exposure of mice *in vivo* or human skin samples *ex vivo* to inflammatory doses of ultraviolet B irradiation induced IL-33 expression within the epidermal and dermal skin layers (34). Proteomic analysis used to determine the extracellular and intracellular roles of IL-33 in primary human endothelial cells revealed the induction of inflammation-related protein expression of the exogenous extracellular IL-33, whereas the knockdown of the endogenous nuclear IL-33 expression had no reproducible effect on the endothelial cell proteome (35).

The results described above support that the expression level and biological role of IL-33 are similar to those of ST2. In general, IL-33 expression is upregulated in inflamed tissues following pro-inflammatory stimulation, and the role of IL-33 in cells may vary under different pathophysiological conditions. In SSc, with an exception during tissue inflammation, the authors proposed that IL-33 commonly responds to tissue injury and typically affects rapid tissue repair and regeneration (36–38).

In patients with SSc, serum levels of IL-33 and sST2 were elevated (39) and positively correlated with the extent of skin sclerosis (higher in diffused cutaneous SSc than in limited cutaneous SSc), severity of pulmonary interstitial fibrosis, and vascular involvement in SSc development (40–44). In the lesion skin tissues, IL-33 expression is altered depending on the disease stage. IL-33 is downregulated in most endothelial cells in early SSc but not in late SSc (45). IL-33 produced by activated dermal fibroblasts/myofibroblasts has been implicated in the fibrotic pathology associated with SSc, which is profoundly increased by hypertrophic and mechanical stress (46, 47).

The expression of IL-33 mRNA is reported to increase in the primary pulmonary fibroblasts from patients with SSc-ILD as well as in those from patients with idiopathic pulmonary fibrosis (IPF). The elevated levels of IL-33 in bronchoalveolar lavage fluids may be useful in differentiating IPF from other chronic ILDs (48). In patients with IPF and SSc-ILD, the expression of full-length IL-33 was elevated in the affected lungs, consistent with the observation reported in a bleomycin-induced mouse model. Under the conditions of ST2 gene deficiency, the full-length IL-33 could stimulate the expression of several non-Th2 cytokines and heat shock protein 70. On the other hand, the matured form of IL-33 was unaffected and instead activated Th2 responses (49). In contrast, the expression of the matured form of IL-33 was enhanced but that of the full-length counterpart reduced in the macrophages of bleomycin-induced mouse lung tissues (50). These findings suggest that the full-length IL-33 may serve as a synergistic pro-inflammatory and pro-fibrotic regulator in the lungs.

## Pathogenesis of IL-33/ST2 in SSC

Fibrosis, a prominent pathological characteristic of SSc (38), is characterized with a deregulated and uncontrolled repair process. Many molecular and signaling pathways involved in the fibrosis of SSc (51, 52), including transforming growth factor (TGF)-β, TLR4, and interferons (IFNs), are well-studied. TGF-β is responsible for both physiological and pathological matrix remodeling (53) as well as fibroblast-myofibroblast transformation (54). TLR4 induces pro-fibrotic responses by activating NF-κB signaling through MyD88, IRAK, and TRAF6. The TLR/NF-κB signaling pathways enhance the TGF-β-dependent fibrotic process (55, 56). IFNs generally act as negative regulators of collagen synthesis and TGF-β-mediated fibrotic responses, while the mechanism of type I IFN signaling in SSc-promoted fibrosis remains unclear (37, 57). The role of IL-33 in SSc was recently evaluated. In pediatric patients with limited cutaneous SSc, high levels of IL-33 and IFN-γ positively correlated with anti-histone and anti-ssDNA antibodies, indicating that the co-expression of IL-33 and IFN-γ may contribute to the pathogenesis of SSc (58). Subcutaneous injection of IL-33 in mice resulted in the development of cutaneous fibrosis, similar to that observed in patients with SSc, including dermal mast cells and skin-infiltrating neutrophils through the suppression of Th1-mediated contact hypersensitivity responses (59). This observation highlights the important roles of IL-33 in SSc. However, the exact mechanisms require further investigation.

Known as a master regulator of pathological fibrosis, TGF-β may be produced by IL-33-induced cells. During the amplification of the alternatively activated M2 macrophage polarization, the IL-33/ST2 pathway was shown to play a significant role (60). IL-33 polarized M2 macrophages to produce IL-13 and TGF-β1 and induced the expansion of type 2 innate lymphoid cells (ILC2s) for the production of IL-13 *in vitro* and *in vivo*. ST2 may protect ILC2s from IL-33 stimulation by reducing the production of IL-5 and IL-13 (61). IL-13 is a well-known profibrotic cytokine downstream of IL-33 in the immune system (51).

IFN-γ may play regulatory roles in physiological processes involving IL-33. In type 2 immune responses, IL-33 and ILC2s are central mediators that promote tissue and metabolic homeostasis, whereas IFN-γ suppresses this pathway and promotes inflammatory responses (62). *In vivo*, the co-expression of IL-33 and IFN-γ in pulmonary fibroblast culture and lungs resulted in the attenuation of IL-33 protein levels (63). IFN-regulated genes may regulate IL-33 gene expression. In both human monocytes and macrophages from C57BL/6 mice, transcriptional activation of the IL-33 gene stimulated by the acute-phase protein serum amyloid A, a TLR2 ligand, may be regulated by IFN regulatory factor 7 (IRF-7) recruited to the IL-33 promoter. Silencing of IRF-7 expression may result in the abrogation of the expression of IL-33 induced by serum amyloid A (64).

In fibrosis, DCs elevated the expression of IL-33 via TLR/NF-κB signaling pathways in response to allergic inflammation, resulting in an increase in the expression levels of MyD88, NF-κB1, NF-κB2, and RelA accompanied with NF-κB p65 nuclear translocation, possibly through a potential autocrine regulation. These elevations may be blocked with a TLR5 antibody or NF-κB inhibitor quinazoline and thought to be decreased in DCs from MyD88-knockout mice (65). The deficiency in the NF-κB negative feedback regulator A20 in hyperactive mast cells may result in amplified pro-inflammatory responses downstream of IgE/FcεRI, TLRs, IL-1R, and IL-33R (ST2), thereby exacerbating inflammatory disorders (66). In addition, Th2-stimulated (allergen-specific IgG immune complexes and house dust mites) signaling occurs through FcRγ-associated receptors on DCs to upregulate IL-33 production and induce Th2-mediated allergic airway inflammation (67).

In conclusion, IL-33 functions as a pro-fibrogenic cytokine in the development of SSc. IL-33 may enhance the TGF-β-dependent fibrotic process by increasing the production of TGF-β and activate TLR/NF-κB-dependent fibrosis signaling pathways, which are regulated by IFN-γ (Table 1).

**Table 1:** Targets/pathways involved in IL-33-dependent fibrosis process.

**Targets/Pathways**	**Effector cells**	**Mediators**	**Role of IL-33**	**References**
TGF-β	M2 macrophages and ILC2s	IL-13, IL-5	IL-33 induced cells to produce TGF-β	(61)
IFN-γ	ILC2s, pulmonary fibroblast, and lungs		IL-33 was inhibited by IFN-γ	(62, 63)
	Monocytes and macrophages	IRF-7	IRF-7 promoted the expression of IL-33	(64)
TLR/NF-κB signaling pathways	Dendritic cells	MyD88, NF-κB1, NF-κB2, and RelA	IL-33 or ST2 was regulated by TLR/NF-κB signaling pathways	(65, 67)
	Mast cells	NF-κB negative feedback regulator A20		(66)

*TGF-β, transforming growth factor-β; ILC2s, type-2 innate lymphoid cells; IFN, interferon; IRF-7, IFN regulatory factor 7; TLR, Toll-like receptor; NF-κB, nuclear factor-κB; IL, interleukin*.

To determine whether IL-33 is a useful therapeutic target, Locksley et al. described the complexity of using IL-33 and therapeutic strategies for altering IL-33 activities *in vivo* (68). The framework of IL-33 biology was described as a stepwise process. First, the focal cellular necrosis or other signals induce the release of IL-33 from the nucleus to maintain homeostasis; IL-33 acts on tissue-resident ST2-expressing effector cells such as ILC2s, regulatory T cells (Tregs), and mast cells to create a tissue environment that limits inflammation and promotes a reparative state characterized by tolerance. Second, amplification occurs upon exposure to chronic stimuli such as allergens and repetitive tissue damage, wherein excess extracellular IL-33 leads to multiple self-stimulating cycles of release to promote chronic allergic pathology, fibrosis, and excess stores of IL-33 in the circulation and tissues. The third step is conversion, wherein the activated inflammatory cells and cytokines responsive to the IL-33/ST2 axis play various roles such as killing pathogens, mounting anticancer immune responses, increasing tissue damage, and repressing the type 2-associated immune regulation responses. In patients with SSc, repetitive tissue damage by other pro-fibrotic mediators in fibroblasts and endothelial cells likely suppresses the IL-33 pool increases and regulatory mechanisms. Next, inflammation is amplified, fibrosis occurs, and tissue IL-33 levels increase, ultimately contributing to tissue fibrosis and sclerosis.

Therefore, IL-33 from different sources can be up- or downregulated to exert pleiotropic roles in SSc. Zhao et al. proposed that these apparently contradictory results indicate the presence of an extremely complex process of IL-33 processing and secretion (69). The functional properties of recombinant IL-33 used in previous studies are becoming well-characterized, whereas the cellular sources of IL-33 in natural and stimulated expression remain largely unknown. Additional studies are warranted to explain the differences between *in vitro* and *in vivo* results.

## Clinical applications of IL-33 in SSC

Various aspects of the clinical applications of IL-33 have been examined. However, few studies have evaluated these effects in patients with SSc. Thus, information may be obtained from studies of other diseases that may be applicable to SSc.

IL-33-responsive ILC2s may promote the restoration of injured skin, lung, and gut cells (70). During the regeneration of injured muscles, fibro-adipogenic progenitor cells are the only known source of IL-33 in muscles. The low level of IL-33 expression in older, injured muscle reduces the recruitment and proliferation effects of non-increased muscle-resident Tregs; after the administration of IL-33, the Treg population increases and regeneration is enhanced (71, 72). Furthermore, the upstream and downstream regulation of the IL-33 gene may promote the remodeling of tissues such as nerves and tendons (73, 74).

In general, studies of IL-33 in patients with SSc have indicated that IL-33 is a novel and important pro-fibrogenic cytokine and a potential biomarker for monitoring disease activity (40–45). Genetic polymorphisms in the IL-33 gene may be useful for the prediction of the risk of various diseases. The IL-33 rs7044343 CC genotype was suggested to be associated with an increased risk of developing SSc and a decreased risk of developing RA; the T allele may be a susceptibility marker for premature coronary artery disease and central obesity and possibly involved in the regulation of IL-33 production (53, 75–77). The first monoclonal anti-ST2 antibody, CNTO-7160, was recently designed as a new IL-33 inhibitor; this antibody is being evaluated in phase I clinical trials for the treatment of severe asthma and atopic dermatitis, but no data have been published to date (78).

## Prospects

The alarmin IL-33 has dual functions of a cytokine and nuclear factor. However, differences in the levels of IL-33 and systemic sST2 indicate intra-individual and inter-individual biological variation, reference changes, and sex-specific differences (79). Moreover, the evaluation of the circulating concentrations of sST2, full-length IL-33, mature IL-33, and complexes of sST2 and IL-33 in the same patients is interesting; measurement of these four analytes and their ratios may increase the understanding of IL-33-related pathophysiology in various diseases (80).

Recent investigations suggested that IL-33 is a novel pro-fibrogenic cytokine in the development of SSc, mainly because it affects the TLR/NF-κB signaling pathways, and TGF-β1 expression is also regulated by IFN-γ. These effects are crucial for the early diagnosis of pulmonary fibrosis. Whether IL-33 is involved in fibroblast activation alone or in combination with other factors is unclear; however, this molecule is likely a potential biomarker and novel therapy target for managing fibrosis in patients with SSc. Furthermore, the inhibitor of IL-33 (CNTO-7160), currently being examined in clinical trials, may possibly be developed as a new therapy for fibrosis in patients with SSc (78).

## Author contributions

LL devised and wrote the manuscript. HZ and XZ revised the manuscript.

### Conflict of interest statement

The authors declare that the research was conducted in the absence of any commercial or financial relationships that could be construed as a potential conflict of interest.

## Abbreviations

ST2L: ST2 longer transmembrane form
sST2: soluble ST2
ILC2: type 2 innate lymphoid cell
Treg: regulatory T cell
DC: dendritic cell
Th1: T helper 1 cell
CD8+ T cell: CD8-positive T cell
iNK T cell: invariant natural killer T cell
NK cell: natural killer cell
SSc: systemic sclerosis
IRAK: IL-1R-associated kinase
TRAF6: tumor necrosis factor receptor associated factor 6
IL: interleukin
ILD: interstitial lung disease
IL-1RAcP: IL-1 receptor accessory protein
NF-κB: nuclear factor-κB
MAPK: mitogen-activated protein kinase
IPF: idiopathic pulmonary fibrosis
TGF: transforming growth factor
TLR: Toll-like receptor
IFN: interferon
IRF-7: IFN regulatory factor 7.
